# Fluorescence Cell Imaging and Manipulation Using Conventional Halogen Lamp Microscopy

**DOI:** 10.1371/journal.pone.0031638

**Published:** 2012-02-08

**Authors:** Kazuo Yamagata, Daisaku Iwamoto, Yukari Terashita, Chong Li, Sayaka Wakayama, Yoko Hayashi-Takanaka, Hiroshi Kimura, Kazuhiro Saeki, Teruhiko Wakayama

**Affiliations:** 1 RIKEN Center for Developmental Biology, Kobe, Japan; 2 Research Institute for Microbial Research, Osaka University, Suita, Japan; 3 Department of Genetic Engineering, Kinki University, Kinokawa, Wakayama, Japan; 4 Laboratory of Animal Reproduction, Graduate School of Agricultural Science, Tohoku University, Sendai, Japan; 5 Graduate School of Frontier Biosciences, Osaka University, Suita, Japan; St. Georges University of London, United Kingdom

## Abstract

Technologies for vitally labeling cells with fluorescent dyes have advanced remarkably. However, to excite fluorescent dyes currently requires powerful illumination, which can cause phototoxic damage to the cells and increases the cost of microscopy. We have developed a filter system to excite fluorescent dyes using a conventional transmission microscope equipped with a halogen lamp. This method allows us to observe previously invisible cell organelles, such as the metaphase spindle of oocytes, without causing phototoxicity. Cells remain healthy even after intensive manipulation under fluorescence observation, such as during bovine, porcine and mouse somatic cell cloning using nuclear transfer. This method does not require expensive epifluorescence equipment and so could help to reduce the science gap between developed and developing countries.

## Introduction

It has long been believed that the excitation of fluorescent vital dyes in cells requires a powerful excitation light source, such as an epifluorescence mercury vapor, Xenon lamp or a laser light, and that a halogen light microscope cannot be used to observe fluorescent images. However, such strong excitation can also cause cellular phototoxicity [1]. This is a particular problem when imaging is prolonged or during continuous exposure to light, such as during embryo micromanipulation. Therefore, several new microscopy systems have been developed to reduce phototoxicity [2], [3], [4]. These do not compromise the viability of cells or embryos because they use a low light intensity for excitation. However, despite their lower phototoxicity, they have complicated mechanisms and are expensive; this makes cell manipulation difficult. Therefore, if fluorescent images could be observed using a conventional transmission microscope with a simple halogen lamp, this would not only reduce phototoxicity but also allow studies in poorly resourced laboratories. In 1971, in contrast to the established systems using high pressure mercury vapor lamps [5], the use of a tungsten halogen lamp source in fluorescence microscopy was anticipated by Heimer and Taylor [6]. Unfortunately, this type of microscopy has not been developed further since that time, probably because of its low excitation efficiency. Moreover, the most serious problem was that the fluorescent and bright field images could not be seen simultaneously in this system, so cell manipulation was impossible. Here, we have developed a new method for the observation of living cells using a conventional transmission microscope that enables both fluorescence and bright field images to be observed simultaneously using a halogen lamp as a light source.

## Results

**Figure 1:**
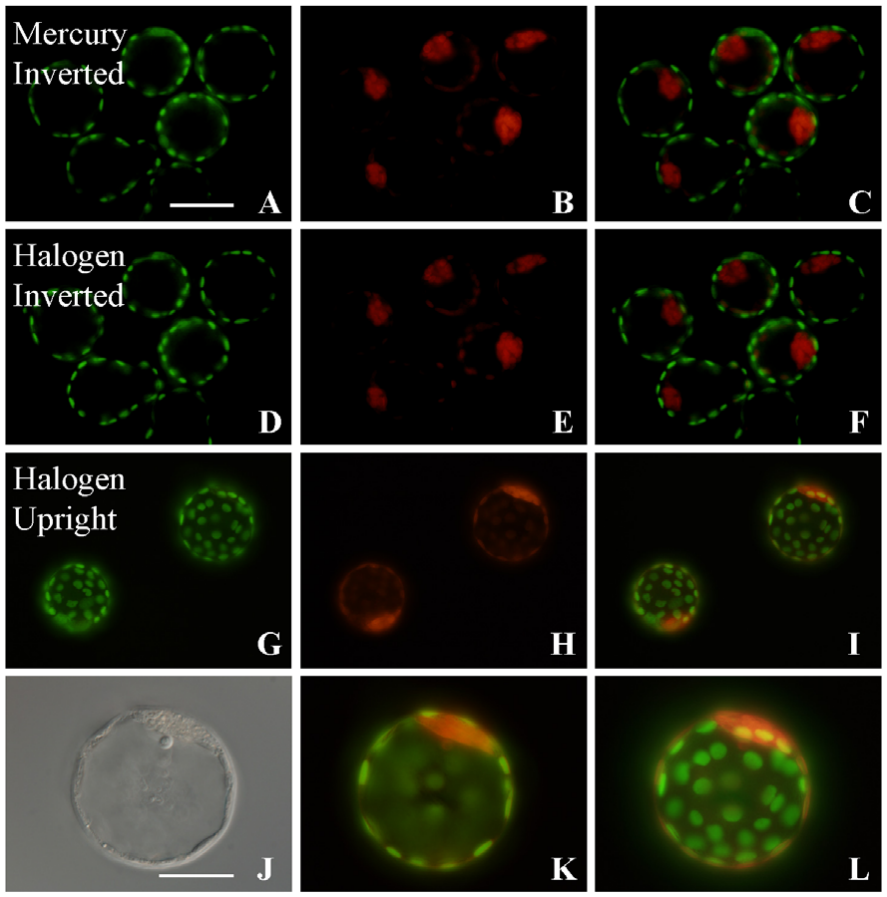
Observation of fluorescent dyes using a halogen lamp. Fixed mouse blastocysts were treated with anti-Cdx2- and anti-Oct3/4-antibodies and stained with Alexa Fluor 488 (green) and 548 (red), respectively, then observed using a mercury vapor lamp (A–C) or an inverted microscope with a halogen lamp (D–F). Although the images produced by halogen light illumination were slightly weaker than images produced with the mercury vapor lamp, they could substitute for those seen using a traditional fluorescence microscope. Similar specimens were observed using an upright microscope with halogen lamp (G–L). These images were taken using an LCPlanF1 objective lens (×20; bar = 100 µm). (J) Bright field illumination. (K, L) A different focal plane of the same embryo shown in (I). (J) Inner cell mass (ICM) cells appear as Oct3/4-positive cells. (K, L) Merged images. (J–L) Observed using an LCPlanF1 objective lens (×40; bar = 50 µm).

To excite and view fluorescence using either an upright or an inverted microscope with a halogen lamp, an excitation filter was placed on the top or bottom of the condenser, respectively, and an emission filter was placed inside the microscope or eyepiece (Figure S1A, B). Because the intensity of a halogen lamp is much less than that of a mercury vapor lamp (Figure S1C, D), all factors affecting the brightness of the halogen lamp were removed from the optical path before observation. Then, to confirm the efficiency of this fluorescence excitation system, doubly stained specimens were used. Mouse blastocysts were immunostained with primary antibodies against Cdx2 and Oct4/Pou5f1 and subsequently labeled with secondary antibodies conjugated to Alexa Fluor 488 and Alexa Fluor 546, respectively. We could detect trophectoderm as Cdx2-positive cells (green, Figure 1A), inner cell mass (ICM) cells as Oct3/4-positive cells (red, Figure 1B) and the merged image (Figure 1C) using an inverted microscope. The fluorescent image was sufficiently bright, even using the middle power setting of the halogen lamp, and was almost as good as images produced with a mercury vapor lamp (Figure 1D–F). Interestingly, the fluorescent image seemed to be similar or brighter with the upright than with the inverted microscope (Figure 1G–L). These images demonstrate that fluorescence observations using a halogen light can be substituted for conventional fluorescence microscopy. However, because the spectrum of light—except for the excitation wavelengths—is blocked by the excitation filter, it has not been possible to see a bright field image simultaneously, which is indispensable for cell manipulation. By contrast, a fluorescence microscope normally contains two light sources (typically a mercury vapor lamp and a halogen lamp) and the signal locality can be detected by switching or combining the images.

**Figure 2:**
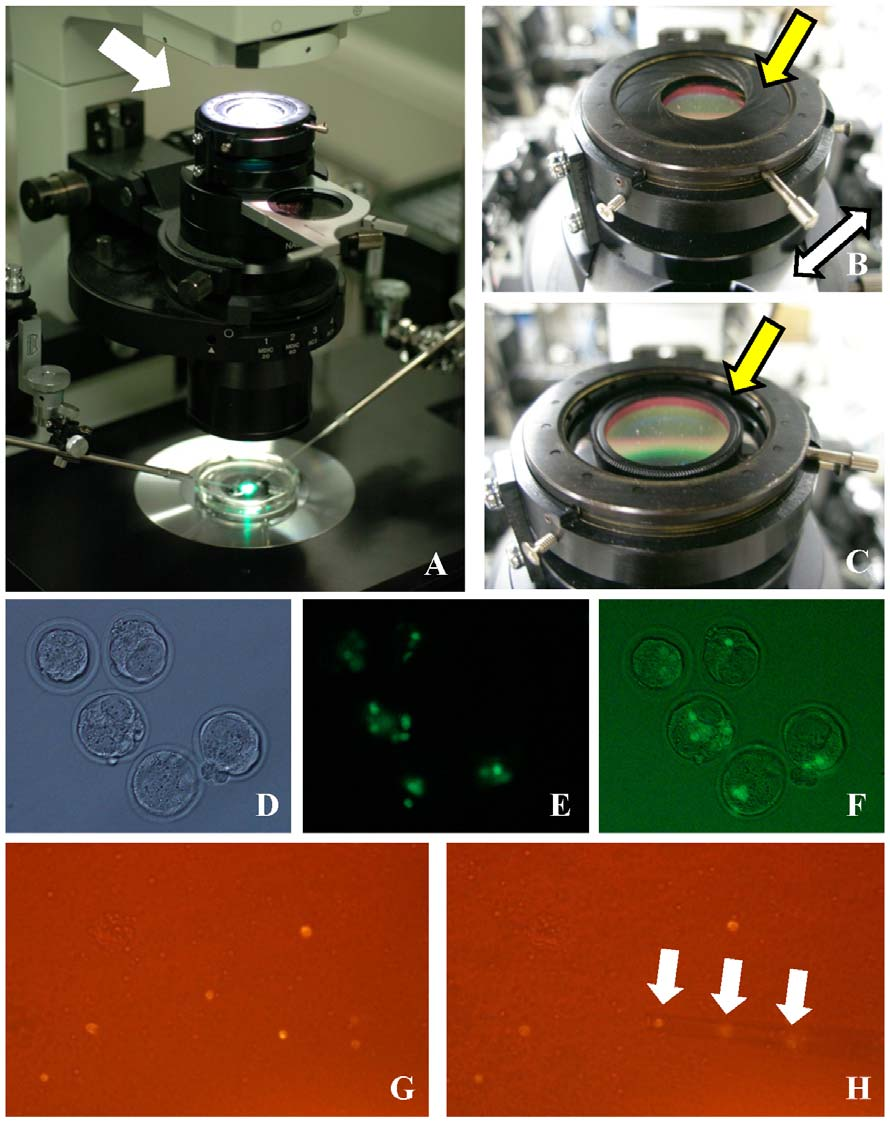
The newly designed filter adapter and its applications. The adapter is placed on the top of the condenser (A). This adapter has a diaphragm (B) and a gap between the filter and frame (C). (D) Bright field and (E) fluorescence of green fluorescent protein (GFP)-labeled embryonic stem (ES) cells in the ICM of a chimeric mouse blastocyst. When the diaphragm is opened, halogen light can pass around the filter and a merged image can be obtained (F). This filter adapter could also be useful in purifying rare cell populations. Typically, fluorescence-activated cell sorting is used for this purpose, but cannot be applied if samples do not contain sufficient cell numbers. We collected spermatogonia from neonatal mouse testes using a CD9 antibody labeled with phycoerythrin. The CD9-positive spermatogonia could be discriminated from other cells and we were able to collect them using a micromanipulator without the need for a mercury vapor lamp. (G) Spermatogonia were detected using a CD9 antibody–phycoerythrin conjugate and positively stained cells (arrows) were drawn into a micropipette using a micromanipulator (H).

To address this shortcoming, we developed an adapter in which the filter is smaller than the optical path and has a diaphragm that allows leakage of light around the periphery (Figure 2A–C). When this diaphragm is closed, all light from the halogen lamp passes through the excitation filter, so that only fluorescence is detected (Figure 2B). However, by opening the diaphragm, the bright field image can be observed simultaneously with the fluorescent signal, and the balance of intensities between fluorescence and bright field pathways can be optimized (Figure 2C). To confirm this approach, we prepared chimeric mouse embryos by injecting green fluorescent protein (GFP)-expressing embryonic stem (ES) cells into the perivitelline space of 4-cell embryos. We then observed the fate of the injected ES cells at the blastocyst stage using an inverted microscope (Figure 2D–F). When the diaphragm was closed, only GFP-expressing cells (ES cell origin) were detected (Figure 2E). However, by adjusting the diaphragm, the non-GFP-expressing cells (host blastomeres) could be observed with the fluorescent signal simultaneously (Figure 2F). Thus, using this filter adapter, it is possible to discriminate the localization of fluorescent positive cells or collect rare cells, such as spermatogonia from the neonatal mouse testis, from others (Figure 2G, H) using bright field illumination.

Finally, we applied this system to cloning by somatic cell nuclear transfer (SCNT). SCNT has a strong potential in the areas of animal reproductive technology and genetic modification in large animals, as well as in regenerative medicine. However, technical difficulties obstruct basic studies and further applications. One example is the removal of metaphase II (MII) chromosomes (enucleation) from oocytes; these are normally obscured in the ooplasm of domestic ruminants by dark lipid droplets. In general, Hoechst dye staining and mercury vapor light observation are commonly used for nuclear staining. However, this dye binds irreversibly to DNA and remains in the cytoplasm (Figure S2), thereby reducing cell or embryo viability [7]. Indeed, when mouse oocytes were treated with Hoechst 33342 dye and exposed to UV light, only 5% of embryos developed to blastocysts after SCNT and strontium activation (Table 1). This toxicity must have been caused either by the Hoechst stain itself or by the intensity of UV excitation. Note that parthenogenetic or intracytoplasmic sperm injection (ICSI)-generated embryos showed more serious damage that the cloned embryos (Table 1) [7]. This was probably because much of the Hoechst dye had been removed from the stained oocytes together with the MII chromosomes during enucleation. Therefore, in traditional enucleation methods, although the position of the first polar body cannot accurately predict the location of the MII chromosomes in most oocytes, a small amount of oocyte cytoplasm near the polar body can be removed assuming that it contains the spindle apparatus [8]. Subsequently, the biopsied cytoplasm is stained with Hoechst dye and the presence or absence of MII chromosomes is checked by fluorescence microscopy to allow the selection of successfully enucleated oocytes. However, because of the blind nature of this approach, there is a risk of removing excessive amounts of ooplasm and compromising the viability of subsequent cloned embryos [9]. Therefore, a combination of fluorescent labeling of the MII chromosomes using a nontoxic dye with our new transmission filter system might help us to perform enucleation more precisely.

**Table 1:** Toxicity of Hoechst and mercury lamp for mouse embryo development.

					In vitro development at 96 h		
Type of embryos	Hoechst staining	Mercury light	No. used oocytes	No. activated/fertilized oocytes	1, 2-cell	8-cell	Blastocyst
Partheno.	−	−	119	87	5 (5.7)	18 (20.7)	64 (73.6)
	+	−	117	47	2 (4.3)	18 (38.3)	27 (57.4)
	−	+	129	117	49 (41.9)	46 (39.3)	18 (15.4)
	+	+	141	62	51 (82.3)	7 (11.5)	0 (0.0)
ICSI	−	−	98	82	8 (9.8)	26 (31.7)	48 (58.5)
	+	−	93	26	7 (26.9)	6 (26.1)	10 (43.5)
	−	+	99	50	6 (12.0)	23 (46.0)	21 (42.0)
	+	+	100	24	13 (54.2)	10 (43.5)	0 (0.0)
Clone	−	−	96	73	62 (85)	55 (75)	38 (52.1)
	+	−	108	76	53 (70)	36 (47)	1 (1.3)
	−	+	114	72	55 (76)	47(65)	24 (33.3)
	+	+	105	79	48 (61)	30 (38)	4 (5.1)

**Figure 3:**
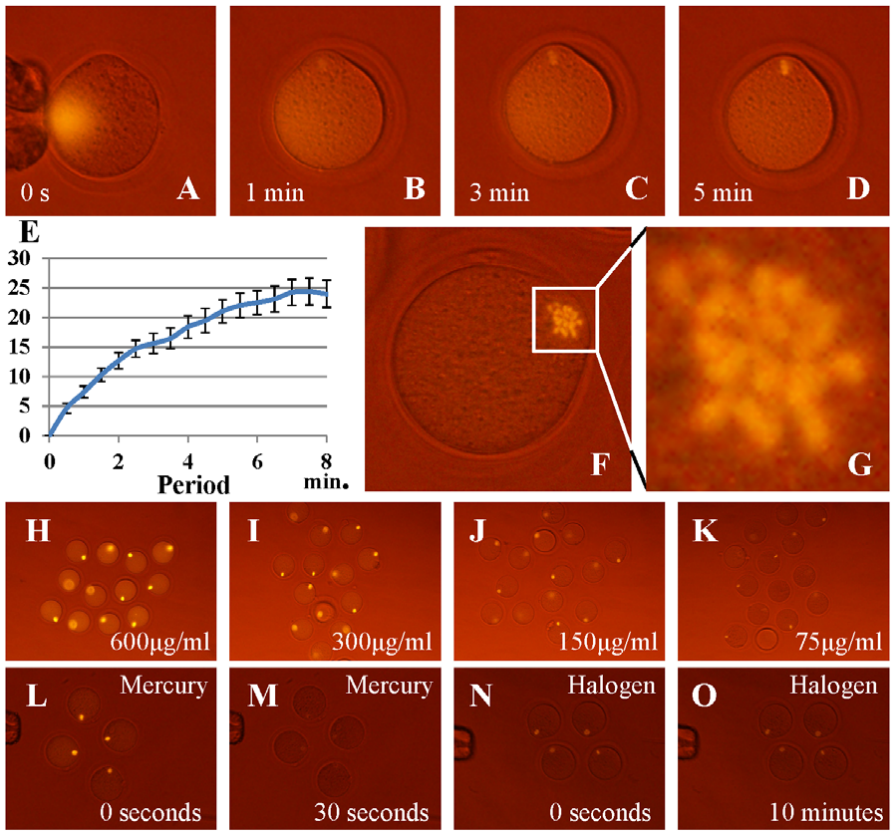
Staining of MII spindle using phycoerythrin and observation. The antibody–phycoerythrin conjugate diffused immediately after microinjection into mouse oocytes (A) but the conjugate could bind to the MII chromosome array within a few minutes (B, C). Five minutes later, the chromosomes became sufficiently bright for enucleation (D). The brightness of MII chromosomes was measured by comparison with the cytoplasm; the luminance increased with time up to 8 min (E). Imaging could reveal individual chromosomes (F, G). Different concentrations of the phycoerythrin conjugate were microinjected into oocytes (H–K). The staining intensity was proportional to the concentration of conjugate used and 75 µg/mL of antibody was the minimum needed for clear observation. Fading of the phycoerythrin label was examined (L–O). (L, M) Images of phycoerythrin-injected oocytes observed using a conventional fluorescence microscope faded within 30 s. (M, N) When these samples were observed using the halogen light system, the image did not fade even when observed continuously for over 10 min.

To deal with this problem, in previous studies we labeled the nucleus with fluorescently labeled antibodies against modified histones and demonstrated that this method showed low toxicity for cells and preimplantation embryos, even after repeated epifluorescence or laser light observations [10], [11]. An antibody directed against the phosphorylated serine 10 of histone H3 (H3S10ph) was shown to bind specifically to M-phase chromosomes. We first examined immunostaining based on anti-H3S10ph labeled with Alexa Fluor 488, Alexa Fluor 555 or Cy3. As a result, all signals could be observed on MII chromosomes using our new filter unit (Figure S3A–F) and these antibodies and dyes did not affect preimplantation embryo development [10]. However, the intensity was not strong enough for enucleating oocytes under bright field illumination. We then chose phycoerythrin as a fluorochrome. This is excited by a wide range of wavelengths and gives a strong fluorescent emission (Figure S1E). When the phycoerythrin-labeled anti-H3S10ph was microinjected into MII oocytes, it dispersed immediately into the cytoplasm (Figure 3A; Video S1). However, the MII chromosomes were clearly visualized within a few minutes (Figure 3B–E). Surprisingly, individual chromosomes could be observed clearly by this system (Figure 3F, G). The brightness was dependent on the concentration of the dye (Figure 3H–K), and when an appropriate concentration was used it did not affect preimplantation embryo development (Table S1). It is noteworthy that although phycoerythrin photobleaches easily, the signal intensity persisted during continuous observation using our filter unit equipped with a halogen lamp (Figure 3L–O). This is probably because a halogen lamp has much lower intensity than a mercury vapor lamp (Figure S1C, D).

**Figure 4:**
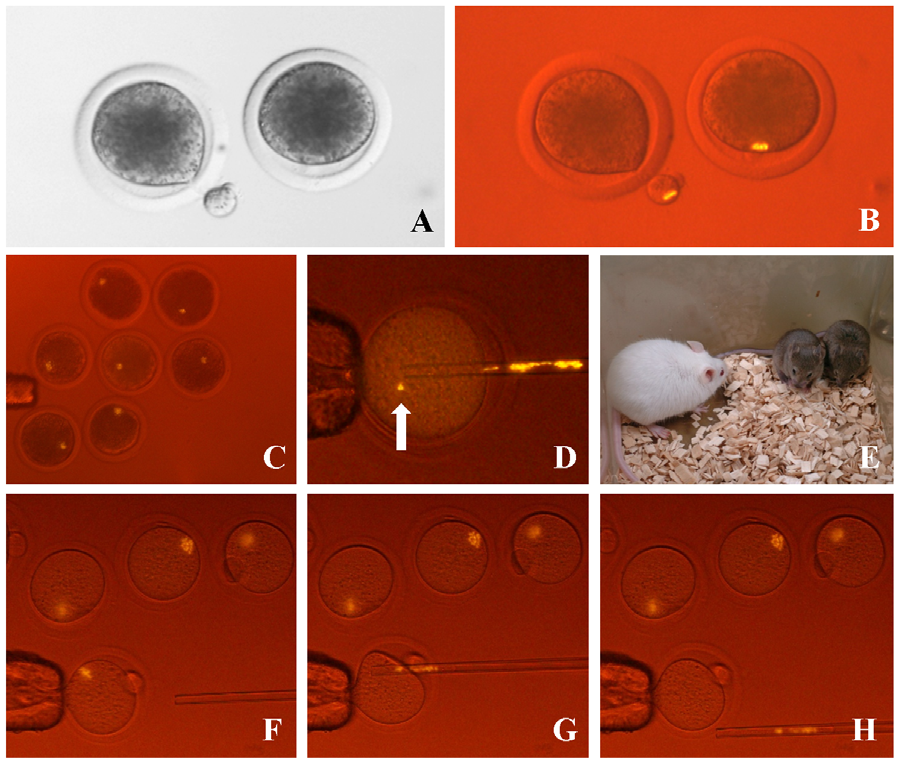
Enucleation of MII chromosomes from oocytes with fluorescence observation using halogen lamp. The MII chromosomes of bovine oocytes are normally invisible by conventional microscopy because of dark lipid droplets in the ooplasm (A). After labeling with an H3S10ph antibody–phycoerythrin conjugate, the MII chromosomes could be recognized clearly and this allowed us to remove them along with minimal cytoplasm (B). MII chromosomes in porcine oocytes were detected by the same method (C). Some chromosomes (arrow) were left occasionally inside the cytoplasm of mouse oocytes after enucleation (D). Live and healthy cloned mice (brown) were obtained after enucleation using this method without any significant decrease in the success rate (E). Enucleation of the MII spindle from mouse oocytes with this system. (F) Before, (G) during and (H) after enucleation.

When applied to bovine oocytes, MII chromosomes were clearly recognized and were easily removed with minimal coincidental cytoplasm removal (Figure 4A, B; Video S2), with a success rate of 100%. The MII chromosomes of porcine oocytes were also seen clearly (Figure 4C). Surprisingly, after enucleation of mouse oocytes, a few chromosomes were left behind in the ooplasm on rare occasions (Figure 4D). If these oocytes were to be used for SCNT, the reconstructed embryo would be aneuploid, and only our system could detect this anomaly. Although we did not record the incidence, the rate was very low. It might depend strongly on the operator's skill; this method would therefore become valuable in laboratories setting out to develop skills in SCNT. In our laboratory, enucleation of oocytes was performed at room temperature. Under these conditions, the spindle might have been partially disrupted leading to misalignment of the chromosomes. If so, using a warmed stage for enucleation might prevent or decrease this chromosomal dispersal.

We next examined the developmental potential of SCNT-generated cloned embryos manipulated using this method. When cloned bovine embryos were cultured for up to 7 days, the blastocyst formation rate was similar to that of controls (26–28% versus 27%) (Table S2). In mouse experiments, we tried to generate cloned offspring because the production of healthy offspring represents the strongest evidence for the quality of the oocytes after experimental manipulation. We were able to obtain many healthy cloned mice with the same success rate as for the controls (Figure 4E; Table 2) [12], [13], which clearly demonstrates that our method did not affect the development of SCNT clones.

**Table 2:** Production of cloned mice using phycoerythrin and antibody injected oocyte.

Conc. of antibody (µg/ml)	No. used oocyte	No. survived after antibody injection	No. survived after enucleation	No. survived after nuclear injection	No. survived after activation	No. pronuclear formation	No. embryo developed to 2-cell	No. offspring (% against pronuclear egg)
0	197	—	197	189	178	170	146	13 (7.6)
75	148	141	140	137	128	117	108	8 (7.0)
150	182	171	169	157	151	146	127	7 (4.8)
300	163	159	157	135	119	115	105	7 (6.0)
Total	690	471	663	618	576	548	486	35 (6.4)

## Discussion

The development of fluorescence microscopy has allowed biologists to observe hitherto invisible cell organelles and large molecules at the normal level of resolution of the light microscope. However, powerful excitation light sources such as mercury vapor lamps or laser lines are used to excite the fluorescent dyes, which can cause cellular phototoxicity [1]. Therefore, several new microscope techniques have been developed not only to improve the resolution of images but also to reduce phototoxicity [2], [3], [4]. However, these expensive microscopes can be used only in a few well-funded institutes; most laboratories, especially in developing countries, do not have even basic fluorescence microscopy. This suggests that the scientific gap between developed and developing countries will continue to widen. Here, we successfully observed several fluorescent dyes and their intracellular targets using a conventional halogen lamp microscope. This system requires only a filter and an adapter to capture fluorescent images, with no need to purchase an expensive mercury vapor lamp or laser unit. This method could open the door for poorly resourced laboratories—including those in poor or developing countries—to participate in advanced biology. Unfortunately, we have to admit that the quality of images in our system cannot match those taken with conventional fluorescence microscopy in terms of optical resolution, as shown in Figure 1.

During these studies, we noted that the fluorescent image was a little brighter with the upright than with the inverted microscope (Figure 1G–L). In this study, as the same objective lens (Olympus, LCPlanFL) was used and as the light power was adjusted to be at the same level in the two systems, this difference in intensity might arise from the different numerical apertures (NA) of the bright field condensers between two microscopes (NA = 0.5 for the inverted; NA = 0.9 for the upright), because the excitation beam passes through the condenser. In addition, as the number of prisms located in the optical path between lens and eyepieces differed between the upright and inverted microscopes, this might also be a reason for the different image intensities.

In the case of SCNT, the MII spindle could be visualized and removed easily following the microinjection of a fluorescently labeled anti-histone antibody into the oocyte. This additional injection treatment required more time than the original SCNT procedure in mice. In addition, oocytes of some species, such as the mouse, are fragile and some oocytes were lysed after this injection. However, in the domestic animal species, oocytes were seldom lysed by the dye injection. If two micromanipulators are available, one can be used to perform the dye injection and the other can be used to perform enucleation at the same time, because the dye stains the MII spindle immediately. Moreover, given the 100% success rate of enucleation (Figure 4F–H), this method makes it unnecessary to confirm the removal of the MII spindle. Therefore, this method of enucleation is significantly simplified and the processing time is reduced compared with conventional approaches.

In education, fluorescence microscopy using vital staining approaches such as the use of GFP is extremely interesting and arouses students' curiosity. However, almost all laboratories in junior and senior high schools use simple microscopes that cannot be used to view such instructive fluorescent images. Using our system will make it possible for all students to have access to advanced fluorescence microscopy without excessive costs. Excitation and bandpass filters are also necessary to observe fluorescent images, and those filters have to be changed based on the type of fluorescent dye used. To observe many fluorescent dyes, a large assortment of filters is required. However, for the purpose of education, one fluorescent imaging technique such as that using GFP is sufficient, and the filter set is not expensive compared with a mercury vapor lamp or laser unit. Thus, access to such a technique will motivate many students to study science and to become scientists.

## Materials and Methods

### Ethics statement

All animal experiments were approved by the Animal Experiment Committee of the RIKEN Center for Developmental Biology (approval no. AH14-13-19). Mice were maintained in accordance with the Animal Experiment Handbook at the RIKEN Center for Developmental Biology. Bovine and porcine ovaries were collected from the Osaka Nanko Zouki and Osaka Shokuniku Zouki slaughterhouses, respectively.

### Animals and oocytes

B6D2F1 and BD129F1 strain mice (SLC, Shizuoka, Japan) were used to provide oocytes, cumulus cells, spermatogonia and fertilized embryos. Surrogate mothers carrying cloned embryos to term were pseudopregnant ICR strain females mated with vasectomized males (SLC). Bovine and porcine oocytes were obtained from ovaries as described [14].

### Antibodies and fluorescent dyes

Blastocysts were fixed as described [7]. For staining blastocysts, rabbit polyclonal anti-Oct3/4 (1∶100 dilution; Santa Cruz Biotechnology, Tokyo, Japan) and anti-Cdx2 (1∶100 dilution; BioGenex, San Ramon, CA, USA) antibodies were used and the embryos were further stained using secondary antibodies. Alexa Fluor 488-labeled goat anti-mouse immunoglobulin (IgG) and Alexa Fluor 546-labeled goat anti-rabbit IgG antibodies were purchased from Molecular Probes (Eugene, OR, USA). For CD9 staining, a phycoerythrin-labeled anti-CD9 antibody was purchased from Santa Cruz Biotechnology. For MII chromosome staining, anti-H3S10ph antibodies labeled with Alexa Fluor 488, Alexa Fluor 555 and phycoerythrin were purchased from Cell Signaling Technology (Beverley, MA, USA). In addition, to examine the appropriate concentration of antibody, we prepared high concentrations of antibody conjugates using the NH2 B-Phycoerythrin labeling kit (Dojindo Molecular Technologies, Japan) according to the manufacturer's protocol.

### Microinjection of oocytes

For oocyte microinjection, Alexa Fluor 488- or 555-labeled anti-H3S10ph antibodies were used undiluted. Phycoerythrin-labeled anti-H3S10ph was used undiluted or at 25–600 µg/mL. About 0.5 µL of the antibody solution was placed in a manipulation chamber. A narrow glass pipette (2–3 µm diameter) was attached to a piezo-activated micromanipulator (Prime Tech, Japan). Once the antibody solution had been aspirated into the pipette, piezo pulses were applied to the oocyte to break the zona pellucida and plasma membrane. A few picoliters of solution were introduced into the oocyte and the pipette was quickly removed. The volume of solution introduced into the ooplasm was controlled by eye, as the ooplasm at the tip of the pipette was pushed away slightly by the emerging solution. Importantly, when we injected the fluorescent dye into the ooplasm, the variation in light intensity in each embryo was very small (approximately 0.8–1.2-fold), suggesting that our microinjection technique was reproducible. This technique was much easier to use than other micromanipulation techniques, such as DNA injection into the pronuclei, ES cell injection into blastocysts, or nuclear transfer. A skillful operator can inject more than 200 oocytes in 1 h, and in our laboratory, the survival rate after antibody injection is nearly 100%.

### Observation of fluorescence through the transmission filter unit using a halogen lamp light source

An inverted microscope (IX-71; Olympus) equipped with a 100 W halogen lamp and a condenser (numerical aperture, NA = 0.5) or an upright microscope (BX53; Olympus) equipped with a 100 W halogen lamp and a condenser (NA = 0.9) were used for imaging. To obtain the highest light intensity, all factors affecting the brightness of the halogen lamp needed to be removed from the optical path before observation. Usually, there is some filter between the condenser and halogen lamp, and these filters greatly reduce the intensity of halogen light. If the condenser itself also includes a filter, such as that found on differential interference contrast (DIC) microscopes, it should also be removed. The diaphragm of the halogen lamp should be open completely. We developed the new filter adapter with excitation filter and diaphragm with support from Olympus, Tokyo, Japan. This filter adapter was a prototype and is not for sale at present, but Olympus plans to make it available commercially soon. To observe GFP and Alexa Fluor 488, a 460–490 nm bandpass (BP) filter was used for excitation and a 510 nm barrier (BA) filter was used to collect fluorescent light. To observe phycoerythrin, Cy3, Alexa Fluor 555 and Alexa Fluor 546, a 480–555 BP filter and 580 nm BA filter were used. BA filters were placed in filter cubes or in the eyepiece. The excitation filter for the mercury vapor lamp and a dichroic mirror were removed from the cube. Because of the strong emission of the phycoerythrin dye, the type of objective lens used for its observation is not important. In this study, an Olympus LCPlanFl 0.4 NA objective lens (×20) and plastic dishes were used for fluorescence observation or enucleation. For observing other dyes, an Olympus UPlanSApo 0.75 NA objective lens (×20) and a glass-bottomed dish were needed.

### Exposure of oocytes to UV light, and parthenogenetic activation or ICSI of mouse oocytes

Mouse oocytes were treated with or without 1 µg/mL of Hoechst 33342 dye and exposed to UV light for 10 s before parthenogenetic activation or ICSI. Before irradiation, the power of the mercury vapor light was adjusted to 100 mW using a U-MWU2 mirror unit with a ×20 objective. After that, mouse oocytes were parthenogenetically activated by strontium as described [15] or live sperm were injected into oocytes by ICSI as described [16]. The oocytes were then cultured in KSOM medium for preimplantation development.

### Nuclear transfer

Nuclear transfer was performed as described [12], [14] except for enucleation. For enucleation, an antibody–dye conjugate was injected into oocytes. Then, any MII chromosomes that were seen were removed using an enucleation pipette (mouse oocytes) or pushed out of the zona pellucida (bovine oocytes). After performing SCNT using those oocytes, cloned embryos were cultured to examine the developmental potential (bovine) or transferred into pseudopregnant ICR females at 0.5 days post copulation (dpc) and live offspring were collected by Cesarean section at 19.5 dpc.

### Production of chimeric embryos

Embryos at the 4- to 8-cell stage were obtained from ICR strain females mated with ICR strain males and GFP-labeled ES cells [17] were injected into the perivitelline space of the embryo. On the following day, chimeric blastocysts were observed using this system.

### Collection of spermatogonia

Testicular cells were dispersed as described [18]. The cell suspension was mixed with an anti-CD9 antibody labeled with phycoerythrin for 20 min, then observed using the fluorescence microscopy system; immunopositive cells were collected using a micromanipulator.

## Supporting Information

Figure S1 Positioning of the newly developed filter adapter, power of mercury and halogen lamp, and phycoerythrin excitation wavelength. (**A**) Inverted microscope with the excitation filter placed on the top of the condenser. (**B**) Upright microscope with the excitation filter placed on the bottom of the condenser and the emission filter left in its original place. (**C**) The mercury vapor lamp produces much more intense emission than the halogen lamp (**D**)(Ref: Tanaka, Takaaki (2003) Fluorescent microscope. in *Kenbikyo no tukaikata note*. (Nojima, Hiroshi ed), Yodosha Japan pp. 71). (**E**) Phycoerythrin can be excited by a very wide range of wavelengths (dotted line, c. 480–570 nm) and therefore a 480–555 nm bandpass filter (blue line) can excite phycoerythrin strongly. The red line shows the barrier filter. The green line shows a dichroic mirror, but there was no need to use it in our system.
(TIF)

Figure S2 Hoechst 33342 dye staining and residual dye in the oocyte cytoplasm. (**A–C**) Intracytoplasmic sperm injection (ICSI)-generated and (**D–F**) SCNT-cloned embryos. (**A, D**) After Hoechst nuclear staining of intact oocytes, MII chromosomes were recognized clearly using fluorescence microscopy. (**B, E**) Even when those oocytes were washed carefully, the residual dye in the oocyte cytoplasm still stained sperm heads or somatic cell nuclei immediately after injection and remained bound to nuclei at the pronuclear or pseudopronuclear stages (**C, F**).
(TIF)

Figure S3 Staining of MII spindle using Alexa Fluor 488, 555 or phycoerythrin, optimal concentration of dye, fading period, and the enucleation using this system. (**A–C**) Alexa Fluor 488; (**D–F** Alexa Fluor 555). Different concentrations of the phycoerythrin conjugate were microinjected into oocytes and imaged using the halogen light system (**G–J**). The staining intensity was proportional to the concentration of conjugate used and 75 µg/mL of antibody was the minimum needed for clear observation. Fading of the phycoerythrin label were examined. (**K, L**) Images of phycoerythrin-injected oocytes observed using a conventional fluorescence microscope faded within 30 s. (**M, N**) When these samples were observed using the halogen light system, the image did not fade even when observed continuously for over 10 min. Enucleation of the MII spindle from mouse oocytes with this system. (**F**) Before, (**G**) during and (H) after enucleation.
(TIF)

Table S1 **Effect of phycoerythrin and antibody for mouse pre-implantation embryo development.**
(DOCX)

Table S2 **Effect of phycoerythrin and antibody for bovine pre-implantation embryo development.**
(DOC)

Video S1 (WMV)

Video S2 (WMV)
