# Supplementary figures and images for: Fluorescence Cell Imaging and Manipulation Using Conventional Halogen Lamp Microscopy
Source: PLoS One. 2012 Feb 8;7(2):e31638. doi: 10.1371/journal.pone.0031638 (PMC3275630; doi:10.1371/journal.pone.0031638)

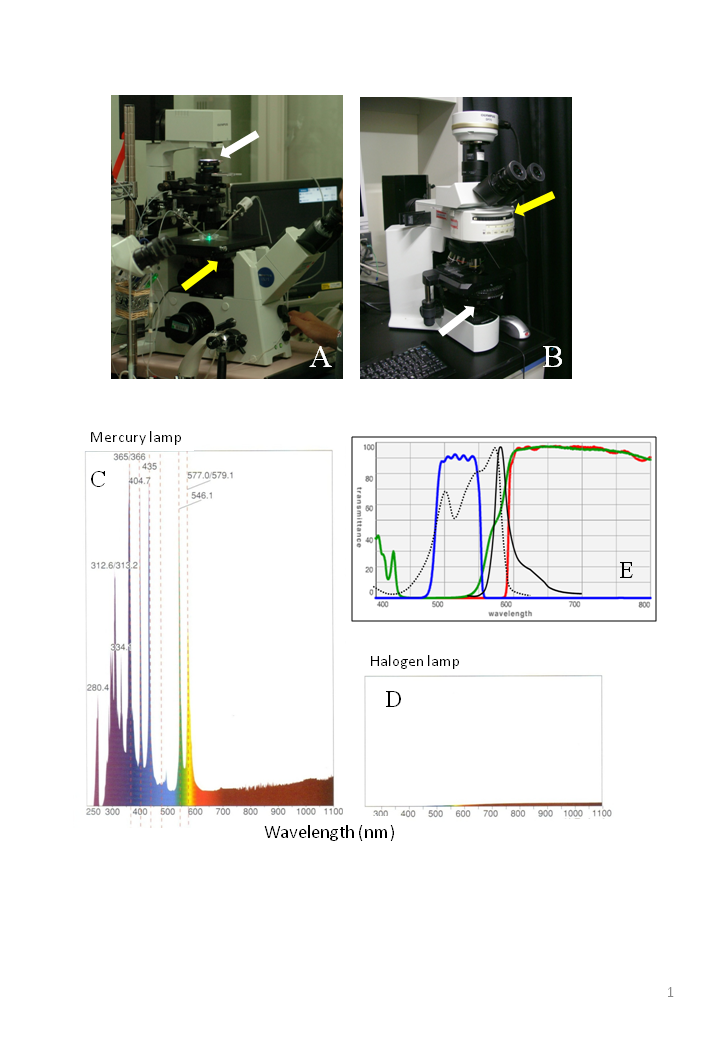

Supplement: Figure S1 — Positioning of the newly developed filter adapter, power of mercury and halogen lamp, and phycoerythrin excitation wavelength. (A) Inverted microscope with the excitation filter placed on the top of the condenser. (B) Upright microscope with the excitation filter placed on the bottom of the condenser and the emission filter left in its original place. (C) The mercury vapor lamp produces much more intense emission than the halogen lamp (D)(Ref: Tanaka, Takaaki (2003) Fluorescent microscope. in Kenbikyo no tukaikata note. (Nojima, Hiroshi ed), Yodosha Japan pp. 71). (E) Phycoerythrin can be excited by a very wide range of wavelengths (dotted line, c. 480–570 nm) and therefore a 480–555 nm bandpass filter (blue line) can excite phycoerythrin strongly. The red line shows the barrier filter. The green line shows a dichroic mirror, but there was no need to use it in our system. (TIF) [file pone.0031638.s001.tif]

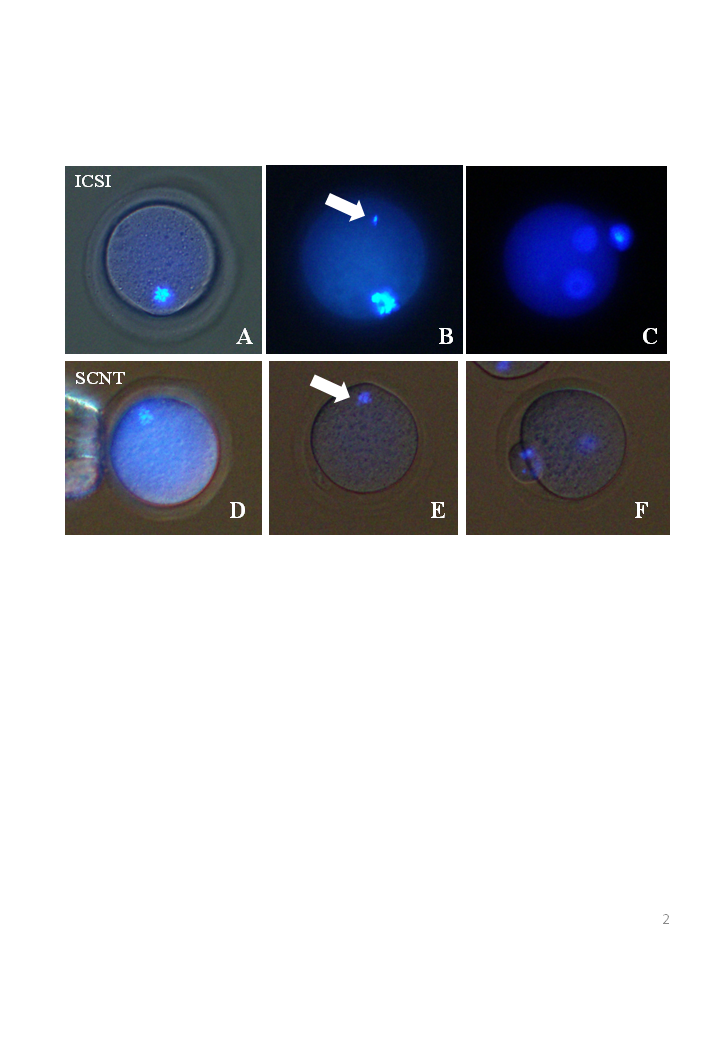

Supplement: Figure S2 — Hoechst 33342 dye staining and residual dye in the oocyte cytoplasm. (A–C) Intracytoplasmic sperm injection (ICSI)-generated and (D–F) SCNT-cloned embryos. (A, D) After Hoechst nuclear staining of intact oocytes, MII chromosomes were recognized clearly using fluorescence microscopy. (B, E) Even when those oocytes were washed carefully, the residual dye in the oocyte cytoplasm still stained sperm heads or somatic cell nuclei immediately after injection and remained bound to nuclei at the pronuclear or pseudopronuclear stages (C, F). (TIF) [file pone.0031638.s002.tif]

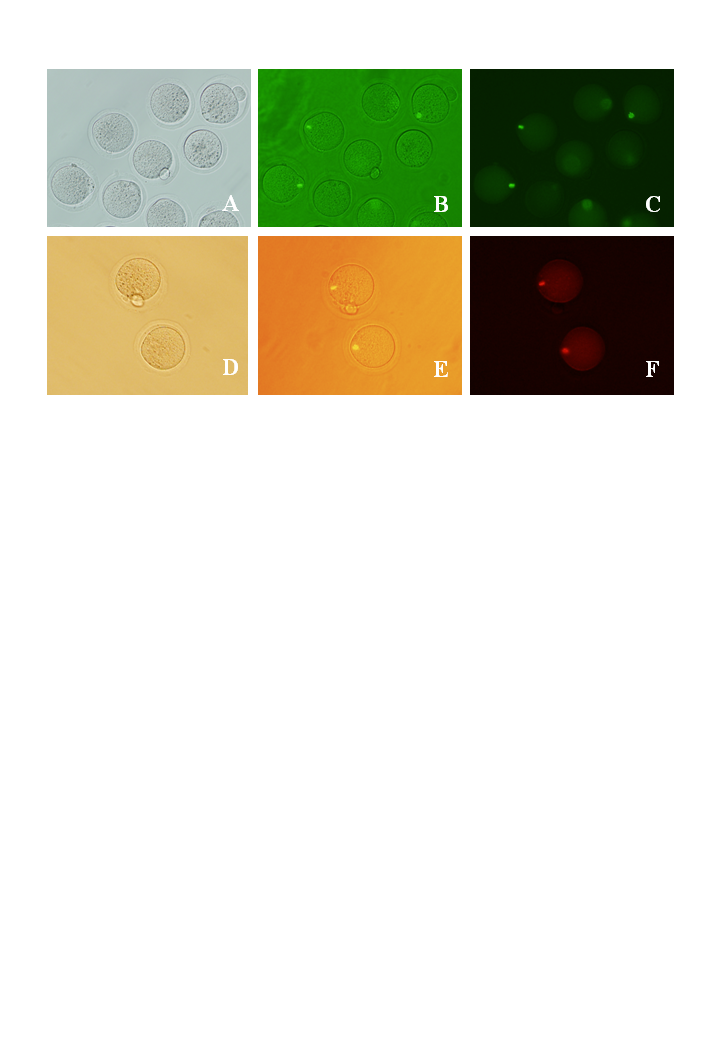

Supplement: Figure S3 — Staining of MII spindle using Alexa Fluor 488, 555 or phycoerythrin, optimal concentration of dye, fading period, and the enucleation using this system. (A–C) Alexa Fluor 488; (D–F Alexa Fluor 555). Different concentrations of the phycoerythrin conjugate were microinjected into oocytes and imaged using the halogen light system (G–J). The staining intensity was proportional to the concentration of conjugate used and 75 µg/mL of antibody was the minimum needed for clear observation. Fading of the phycoerythrin label were examined. (K, L) Images of phycoerythrin-injected oocytes observed using a conventional fluorescence microscope faded within 30 s. (M, N) When these samples were observed using the halogen light system, the image did not fade even when observed continuously for over 10 min. Enucleation of the MII spindle from mouse oocytes with this system. (F) Before, (G) during and (H) after enucleation. (TIF) [file pone.0031638.s003.tif]
