# Supplementary material for: Fluorescence Cell Imaging and Manipulation Using Conventional Halogen Lamp Microscopy
Source: PLoS One. 2012 Feb 8;7(2):e31638. doi: 10.1371/journal.pone.0031638 (PMC3275630; doi:10.1371/journal.pone.0031638)
Supplement: Table S1 — Effect of phycoerythrin and antibody for mouse pre-implantation embryo development. (DOCX) [file pone.0031638.s004.docx]

**Table S1.** **Effect of phycoerythrin and antibody for mouse pre-implantation embryo development**

| Conc. of antibody (µg/ml) | No. used oocytes | Survived after  antibody injection | Survived  after ICSI | Pronuclear formation | No. development at 96h | | | |
| --- | --- | --- | --- | --- | --- | --- | --- | --- |
|  |  |  |  |  | 1-cell | 2-cell | 4,8-cell | Morulae /Blastocyst |
| 0 | 117 |  | 74 | 74 (100.0) | 1 (1.4) | 2 (2.7) | 2 (2.7) | 69 (93.2) |
| 75 | 88 | 75 | 52 | 51 (98.1) | 0 (0.0) | 4 (7.8) | 4 (7.8) | 43 (84.3) |
| 150 | 90 | 80 | 54 | 46 (85.2) | 3 (6.5) | 4 (8.7) | 9 (19.6) | 30 (65.2) |
| 300 | 84 | 79 | 48 | 45 (93.8) | 4 (8.9) | 13 (28.9) | 10 (22.2) | 19 (42.2) |
