# Supplementary material for: Fluorescence Cell Imaging and Manipulation Using Conventional Halogen Lamp Microscopy
Source: PLoS One. 2012 Feb 8;7(2):e31638. doi: 10.1371/journal.pone.0031638 (PMC3275630; doi:10.1371/journal.pone.0031638)
Supplement: Table S2 — Effect of phycoerythrin and antibody for bovine pre-implantation embryo development. (DOC) [file pone.0031638.s005.doc]

**Supporting information**

**Table S2.** Effect of phycoerythrin and antibody for bovine pre-implantation embryo development

| Concentration of antibody (µg/ml) | No. enucleated oocyte | No. reconstructed (%) | No. cultured embryo | No. cleaved embryos（%） | No. blastocyst （%） |
| --- | --- | --- | --- | --- | --- |
| 0 | 96 | 82(85) | 79 | 56(71) | 15(27) |
| 150 | 97 | 70(72) | 62 | 38(61) | 10(26) |
| 300 | 87 | 73(84) | 65 | 40(62) | 11(28) |
